# Beyond CD19: Opportunities for Future Development of Targeted Immunotherapy in Pediatric Relapsed-Refractory Acute Leukemia

**DOI:** 10.3389/fped.2015.00080

**Published:** 2015-10-01

**Authors:** Haneen Shalabi, Anne Angiolillo, Terry J. Fry

**Affiliations:** ^1^Center for Cancer and Blood Disorders, Children’s National Medical Center, Washington, DC, USA; ^2^Hematologic Malignancies Section, Pediatric Oncology Branch, Center for Cancer Research, National Cancer Institute, National Institutes of Health, Bethesda, MD, USA

**Keywords:** chimeric antigen receptor, immunotherapy, adoptive, relapsed leukemia, chimeric antigen receptor safety, pediatric acute leukemia

## Abstract

Chimeric antigen receptor (CAR) T cell therapy has been used as a targeted approach in cancer therapy. Relapsed and refractory acute leukemia in pediatrics has been difficult to treat with conventional therapy due to dose-limiting toxicities. With the recent success of CD 19 CAR in pediatric patients with B cell acute lymphoblastic leukemia (ALL), this mode of therapy has become a very attractive option for these patients with high-risk disease. In this review, we will discuss current treatment paradigms of pediatric acute leukemia and potential therapeutic targets for additional high-risk populations, including T cell ALL, AML, and infant ALL.

## Introduction

From 1975 to 2010, the overall incidence of pediatric cancer in the US has increased slightly, by an average of 0.6% per year (1). Acute lymphoblastic leukemia (ALL) remains the most commonly diagnosed malignancy in pediatrics, with approximately 2,900 new cases/year in the United States (1). With intensive multi-drug chemotherapy children with standard risk (SR) B-precursor ALL, as defined by the NCI/Rome criteria (age 1–9.99 years and initial white blood count <50,000/μL), have cure rates of ~90% (2). However due to its high incidence, acute leukemia remains the leading cause of death in pediatric patients with cancer. Recent clinical trials have risk-stratified patients based on cytogenetics, clinical variables [central nervous system (CNS) and testicular status], and early treatment response (3). Children’s Oncology Group (COG) retrospectively reviewed more than 6,000 pediatric patients with newly diagnosed ALL and adopted a four-group classification after induction therapy: low-risk, average-risk, high-risk, and very high-risk (4). The 4-year event-free survival (EFS) varied intensely among the four groups, 91, 86, 76, and 46%, respectively, demonstrating the need to identify high-risk patients earlier, and alter therapy accordingly, including the incorporation of novel treatment approaches for those patients in the highest risk categories (5). This risk-adapted classification aims to give patients the best chance at cure while sparing unnecessary toxicity.

The reality of relapse and/or refractory disease has been a challenge for oncologists as toxicity of conventional therapy has limited dose escalation, and EFS for these high-risk patients remains suboptimal. Relapse of ALL is defined as disease recurrence at any site after a period of time in which the disease was in complete remission. Refractory disease is defined as inability to achieve a complete remission despite multiple induction attempts. After relapse, chance of cure significantly diminishes due to acquired mechanism of resistance and poor treatment tolerance. With first line chemotherapy intensified to induce a remission, leukemic blasts that are present at relapse have been exposed to the most effective drug regimens and have gained mechanisms that help them survive these cytotoxic agents (6). With a combination of chemotherapy and hematopoietic stem cell transplant (HSCT), 30–50% of all children with relapsed ALL can be cured albeit with the potential for substantial morbidity (7, 8). Results from the Children’s Cancer Group (CCG) 1941 marrow relapse study showed that 50% of patients failed to enter remission, died from toxicity, or relapsed again after achieving a brief second remission (9). Prognostic factors associated with relapse leading to poor outcome include time to relapse, site of relapse, cytogenetics, and immunophenotype [T cell ALL, infant ALL, acute myeloid leukemia (AML)] (6, 10). This review will focus on acute leukemia in pediatrics and discuss current treatment paradigms and potential therapeutic targets for immunotherapy.

## Acute Leukemia Subtypes in Pediatrics

### B cell leukemia

Although the majority of patients diagnosed with pre-B cell ALL are cured, patients with certain prognostic indicators known thus far, such as early relapse, unfavorable cytogenetics, hypodiploidy, persistent minimal residual disease (MRD), have more challenges achieving and sustaining a remission. Pediatric Oncology Group (POG) 9061 aimed at treating patients with isolated CNS relapse with intensive chemotherapy and delayed radiation therapy (RT). The study showed improved outcomes with a delay in RT by 6 months, so more intense systemic chemotherapy could be administered. The 4-year EFS for patients with a complete first remission (CR1) ≥18 months was 84 ± 5% compared to 46 ± 10% for those with CR1 <18 months (*p* = 0.0002) (11). In support of these data, the results of POG 9412 also demonstrated better outcome for those patients with isolated CNS relapse with CR1 ≥18 months with a 3-year EFS of 77 ± 7% compared to 52 ± 11% EFS for those with CR1 <18 months (*p* = 0.0267) (12). Historically, patients with bone marrow relapse, early and late, have had challenges entering a CR2 and have had poor overall outcomes. COG study AALL01P2 looked at inducing a CR2 in patients with early (<36 months from initial CR) or late (>36 months from initial CR) medullary relapse with higher intensity induction chemotherapy. The 4-month EFS for early and late isolated B-precursor marrow relapse were 62 ± 6% (*n* = 72) and 93 ± 4% (*n* = 55), respectively (13). These results are comparable to previous CCG and St. Jude studies. Analysis of relapsed HR-ALL patients treated with either standard or augmented post induction therapy on CCG 1961 demonstrated that 3-year post-relapse survival (PRS) was 30 ± 3.7% (CR1 <36 months) vs. 57.8 ± 6.4% (CR1 ≥36 months), *P* < 0.0001 (14). Also it showed that patients with isolated CNS relapse had better PRS than those with medullary relapse, 52.2 ± 7.4 and 29.7 ± 3.7%, respectively (*P* < 0.001) (14). Similarly, St. Jude evaluated 106 patients with isolated or combined marrow relapse disease and saw a second remission in 66% of early relapse patients vs. 81% of late relapse patients. The 5-year EFS estimates for patients with early relapse (CR1 <36 months) were 12.5 ± 3.9 vs. 42.6 ± 7.8% for patients with late relapse (CR1 ≥36 months) (15). Collectively, these results demonstrate that early and isolated medullary relapse are poor prognostic indicators for patients.

Historically, patients diagnosed with Philadelphia chromosome-positive (Ph+) ALL (BCR–ABL fusion) have had poor outcomes. However, with the discovery of tyrosine kinase inhibitors (TKI), this high-risk subgroup has transformed into a leukemia that is curable. In childhood Ph+ ALL, imatinib mesylate, one of the first TKI, has shown tremendous improvements in 5-year EFS when combined with chemotherapy, 70 ± 6 vs. historical controls with chemotherapy 35 ± 4% (16). Genetic studies over the last decade have identified a Ph-like subtype of leukemia that also has dismal outcomes in pediatric patients with ALL. This group does not have the typical BCR–ABL fusion protein, but shares a similar gene-expression profile to Ph+ ALL (17). It is characterized by deletions in genes that are involved in B cell development and signaling pathways, *IKAROS, PAX5, E2A*, among others, and has shown resistance to standard chemotherapy (18). In a retrospective review of 1,700 previously diagnosed patients with pre-B cell ALL, 15% of these patients were identified as having Ph-like ALL based on genetic studies. Comparative analysis showed that the 5-year EFS and OS among patients with Ph-like ALL were inferior to children with other HR B cell ALL, 58.2 ± 5.3 vs. 83.9 ± 1.5% and 72.8 ± 4.8 vs. 92.1 ± 1.1%, *P* < 0.001, respectively (17). This trend was also noted in adolescent and young adult patient populations. Further studies in this subpopulation are needed to determine if small molecule inhibitors in combination with standard chemotherapy can improve remission rates.

Another significant prognostic indicator in children with pre-B ALL is blast cell ploidy. Hypoploidy is defined as a karyotype that has fewer than 45 chromosomes, and has been associated with poor prognosis in pediatric patients with ALL. Approximately 6% of newly diagnosed B cell ALL patients have hypoploidy, which is comprised of high-hypodiploid (45 chromosomes), low-hypodiploid (36–44 chromosomes), and near-haploid (25–29 chromosomes) (19). Most patients in this category have blast cells with 45 chromosomes, with approximately 1% of patients having fewer than 44 chromosomes. In a retrospective review performed at St. Jude Children’s Research Hospital, 979 newly diagnosed patients with ALL underwent a complete chromosomal analysis and 6.8% of patients were identified as having hypoploidy (19). Results of this study showed that patients with high-hypodiploid had outcomes similar to SR patients; however, patients with blast cells <45 chromosomes had significantly inferior 5-year EFS, 74.9 ± 1.6 vs. 20 ± 10.3%, *p* < 0.001, respectively (19). In another retrospective collaborative review, 130 cases of low-hypodiploid (<44 chromosomes) were analyzed and results were similar to the St. Jude experience, showing that patients with more than 44 chromosomes had better EFS and OS than those with <44 chromosomes (5-year EFS 52.2 ± 9.3 vs. 30.1 ± 7.0%, *P* = 0.01) (20). A recent paper by Mehta et al. described 78 pediatric patients with hypodiploid ALL who underwent a myeloablative HSCT from 1990 to 2010 and showed that those children with fewer than 43 chromosomes had higher mortality and treatment-related failures. The 5-year OS for patients with ≤43 chromosomes adjusted for disease status and transplant time period was 38 vs. 71% for those with ≥44 chromosomes, *p* = 0.01 (21). Thus, more therapeutic studies are needed for this patient population in order to achieve higher remission rates.

Minimal residual disease has been shown to be an important prognostic marker in patients with ALL. A prospective study by Coustan-Smith et al. looked at MRD in 195 newly diagnosed pediatric patients with ALL who received induction therapy. MRD positivity was defined as those patients who had ≥0.01% of leukemia blasts identified by flow cytometry. This study noted a significant difference in the 5-year cumulative incidence of relapse of patients who had MRD-positive disease as compared to those children who were MRD-negative after induction therapy, 43 ± 21 vs. 10 ± 3%, *P* < 0.001 (22). Additionally, a prospective study performed by COG assessed 1971 pediatric patients after induction therapy. Patients who had MRD ≥0.01% had a 5-year EFS of 59 ± 5%, as compared to those who were MRD-negative at the end of induction, 88 ± 1%, *P* < 0.0001 (23). Intensification of therapy is currently the standard of care for those patients with ALL that have day-29 MRD ≥0.01%. In one recent study, Borowitz et al. showed that intensifying chemotherapy based on MRD positivity after induction can improve EFS transiently; however, OS and 3-year EFS did not differ (24). Continued trials are ongoing to determine if this upstaging improves outcomes for this patient population.

In multiple retrospective reviews, MRD appeared to contribute significantly to outcomes in pediatric patients after HSCT. In one prospective study, 25 patients had MRD testing prior to HSCT, with 17 of these patients having MRD-negative disease. Six out of eight patients with MRD-positive disease relapsed post HSCT as compared to one in the MRD-negative cohort, *P* < 0.0001 (25, 26). Additionally, in a recent study performed by COG and the Pediatric Blood and Marrow Transplant Consortium (PBMTC), 144 patients with HR-ALL underwent HSCT and results of this study demonstrated that patients who entered transplant with MRD ≥0.1% disease had a higher risk of relapse after transplant, HR 3.3, *P* = 0.01 (27). The importance of MRD prior to transplant has been emphasized in a recent publication from this study where next generation sequencing of the immunoglobulin V(D)J region was used to identify MRD at very low levels, with no relapses occurring in the group that was MRD-negative using this ultra-sensitive method (28). Although the use of conventional chemotherapeutic agents remains the mainstay of treatment for pediatric patients with acute leukemia, those patients with persistent MRD-positivity represent an extremely high-risk group that may benefit from novel therapeutic approaches in order to eliminate their leukemia burden (26).

Several trials have been performed in order to determine what the best treatment course for patients with high-risk acute leukemia should be and results are inconclusive. In a study performed by Leung et al., patients with HR-ALL and AML had favorable outcomes after HSCT (29). Transplant is thought to be more effective than chemotherapy alone due to at least two different factors: myeloablative conditioning and graft vs. leukemia (GVL) effect. The use of total body irradiation as part of the myeloablative conditioning is generally used in patients with ALL to facilitate better engraftment and to more effectively eliminate any leukemia cells that remain prior to transplant (30, 31), however, whether this TBI-based conditioning is more effective than chemotherapy only preparative regimens has not been proven in randomized trials. Nonetheless, the American Society for Blood and Marrow Transplantation (ASBMT) recommends myeloablative TBI regimens for all patients with ALL undergoing HSCT based on non-randomized data suggesting better survival outcomes (32). GVL effect has been confirmed as a contributor to the curative potential of allogeneic HSCT for leukemia mediated primarily by donor lymphocytes (T cells and natural killer cells) that can control MRD and maintain remission. Although the potency of GVL for ALL has been challenged, in the above-mentioned COG and PBMTC trial, ASCT0431, patients were randomly assigned to standard GVHD treatment arm, tacrolimus/methotrexate, vs. the experimental arm, sirolimus/tacrolimus/methotrexate. The addition of sirolimus did decrease Grades 2–4 acute GVHD significantly in patients but did not improve patient survival. It was noted that those patients who developed Grade 1–3 acute GVHD had an improved EFS, HR 0.5, *P* = 0.02, suggesting that the GVL effect can confer a survival advantage in ALL (27).

In a prospective study of children diagnosed with very high-risk ALL, as defined by refractory disease, t(9;22) or t(4;11) clonal abnormalities, and prednisone-poor response (PPR) associated with T immunophenotype and/or WBC count >100 k/mcL, patients who underwent a related donor HSCT had an improved 5-year EFS as compared to those treated with chemotherapy alone, 56.7 ± 5.7 vs. 40.6 ± 3.1%, *P* = 0.02 (33). Berlin–Frankfurt–Munster (BFM) group and Associazione Italiana Emotologica Oncologia Pediatrica (AIEOP) have evaluated patients with HR-ALL in first remission and compared their OS with chemotherapy alone vs. HSCT. HR criteria in this study included MRD-positive disease at day 78, induction failure, t(4,11) translocation, and PPR. There was no statistically significant difference in EFS for patients given HSCT or chemotherapy alone when adjusted for time waiting to HSCT (34). Thus, studies on the role for HSCT in ALL have been conflicting but most agree that this procedure can offer an advantage for at least some high-risk patients (35, 36).

Despite the collaborative efforts of groups worldwide, infants with ALL have notably inferior outcomes then older children. Traditionally, infants (<365 days old) were treated on HR protocols with no specificity in regimen; however, this resulted in suboptimal outcomes, 3-year EFS ~30% (37). Cooperative trial groups, CCG and POG, demonstrated that intensified systemic chemotherapy with the addition of intrathecal chemotherapy led to improved overall survival as compared to previous studies (50 vs. 43%) (38). However, overall relapse rate remained a major concern, with most relapses occurring within 1 year (39). Analysis from multiple cooperative study groups identified several prognostic factors that were associated with poor outcomes: age <6 months, WBC count >50 k/mcl, MLL rearrangement, and CD10 negativity (38–44). Japanese studies were among the first to report differences in outcome for patients with germline MLL as compared to those with MLL-rearranged disease. One hundred and two infants were enrolled on two Japanese studies, MLL96/98, with results showing dramatic differences; 22 patients with germline MLL had 5-year EFS and OS of 96% vs. 80 infants with MLL rearrangement having a 5-year EFS and OS of 39 and 51%, respectively (42–44). The Interfant Study Group, which comprises the largest worldwide collaboration devoted to infant ALL research, also found that infants with germline MLL had the best outcome, with a 5-year EFS 74% (40). Additionally, for patients treated on Interfant-99, prednisone-good responders had more superior 4-year EFS than those with prednisone-poor response, 56.4 vs. 29.8%, respectively. Nevertheless, the majority of patients with infant ALL continue to have poor outcomes. HSCT remains controversial in this subgroup, with some studies showing no clinical benefit, and in fact some showing worsened outcome in infants with ALL after transplant (45). Interfant-99 showed no significant difference in EFS comparing patients who received chemotherapy vs. HSCT (40).

Flt3 kinase is an important oncogene in adult and pediatric AML and has recently become an area for targeted therapeutics in infant ALL. Gene-expression studies have shown high levels of Flt3 in leukemia blasts in infants and children with MLL-rearranged leukemia (46). Preclinical data have shown that Flt3 kinase inhibitors have *in vitro* effects, with most pronounced response in samples with Flt3 overexpression. The current COG protocol incorporates the above prognostic factors and classifies patients into three groups based on MLL rearrangement and age. In addition to traditional backbone therapy for infant ALL, this study, AALL0631, is the first trial to incorporate novel targeted therapy, Flt3 inhibitor- CEP-701 (lestaurtinib) (47, 48). Results of this study are maturing, however hold promise that small molecule inhibitors can improve outcomes for infant ALL.

### T cell leukemia

Historically, patients diagnosed with T cell ALL had worse prognoses than their counterparts with B cell ALL (49). Some reasons for this include higher WBC count at presentation, increased induction failure, and increased risk of CNS relapse. With the implementation of high intensity induction regimens, the EFS has increased significantly over the last three decades, from 15–20 to 50–85% (49). In a retrospective review done by Dana Farber Cancer Institute (DCFI), patients with T cell ALL were 8.3 times more likely to have an induction failure, and were 2.7 times more likely to have CNS relapse than those patients with B cell ALL. Despite the significant advances in treatment of T cell ALL, those who have relapsed disease have dismal outcomes with 3-year EFS reported at <15% (9, 13, 50). In the COG study AALL01P2, only 2/7 patients with T cell ALL went into a CR2 and no patients with T cell ALL survived >1 year from relapse (13). In the BFM-ALL 87 relapse study, patients with T cell phenotype had an exceptionally poor outcome, with a 10-year EFS probability estimated at 15 ± 1.0% (*P* < 0.001) (50). Thus, novel treatment approaches for relapsed T cell ALL are clearly needed.

The existence of a more immature phenotype of T cell ALL has been identified in the last decade. Early T cell precursors (ETP) are thymocytes that have a distinct immunophenotype (CD1a^−^, CD8^−^, CD5^dim^, +myeloid/stem cell marker) and are thought to belong to very primitive T cells that have retained multi-lineage differentiation potential (51). Patients classified as having ETP-ALL based on immunophenotyping have done significantly worse than more mature T cell phenotype ALL, one review noting a 10-year OS for patients with ETP-ALL of 19 vs. 84% for those with more mature T cell ALL (51). COG study AALL0434 looked prospectively at patients with T cell ALL and categorized them into ETP (11%), near-ETP (those with elevated CD5, 17%), and not-ETP (72%) based on flow cytometry. In a provocative interim analysis, contrary to most prior reports, all three groups showed excellent 5-year EFS and OS that were not statistically significant: ETP (87, 93%), near-ETP (84, 92%), and not-ETP (87, 92%) using the treatment approach employed in this protocol (52). However, they did note that patients with ETP and near-ETP had significantly higher rates of induction failure, 7.8 and 6.7%, respectively, vs. those with not-ETP 1.1% (*P* < 0.0001) (52). Additionally, this study is evaluating whether purine nucleoside analog nelarabine will improve outcomes for T cell ALL when combined with front-line chemotherapy. Nelarabine is a water-soluble prodrug of 9-B-arabinofuranosylguanine (araG) and inhibits DNA synthesis by causing accumulation of araG nucleotides in T cells (53, 54). In a phase II pediatric trial with patients receiving single agent nelarabine, 106 patients were evaluated and risk-stratified into four groups based on first or second relapse with >25% blasts in bone marrow (excluding CNS disease), CNS disease, or those with <25% relapse in marrow. Response rate was greatest for patients with first relapse, 55%, with 16 CR and 2 partial remission (PR) (54). These results hold promise for patients with relapsed T cell ALL as their overall prognosis is poor with conventional therapies, and also could be an option for T cell ALL patients as upfront therapy.

### Acute myeloid leukemia

Acute myeloid leukemia accounts for 20% of newly diagnosed acute leukemia in pediatrics and, with the exception of acute promyelocytic leukemia, confers a worse prognosis then ALL (1). Survival rates for AML have increased dramatically since 1960 with the introduction of more aggressive induction chemotherapy and more intensified regimens. Today, pediatric patients diagnosed with AML have ~50–60% overall survival (55). Through the efforts of multiple cooperative studies, numerous poor prognostic features have been identified; high WBC count, monosomy 5 or 7, 5q deletion, FLT3-internal tandem duplication (ITD) with high allelic ratio, and patients with MRD at the end of the first induction cycle (55–62). In the United Kingdom, the Medical Research Council (MRC) AML trial 10 demonstrated that response rate after course 1 of induction was highly predictive of outcome, with those achieving CR having an OS of 53%, as compared to those with PR, OS 44%, and those with resistant disease, OS 22% (*P* < 0.0001) (56). Treatment with current chemotherapy regimens has reached a plateau, as toxicities will likely preclude further increases in intensity. This emphasizes the need for more targeted therapies in high-risk pediatric AML.

Flt3 overexpression has been identified as a recurring feature of multiple leukemias, and, as seen in infant ALL, has created a lot of interest for targeted therapy in AML. Abnormalities in the Flt3 gene occur in two ways in AML, point mutations in the tyrosine kinase domain and ITDs, which are replicates of three nucleotides that maintain an intact reading frame without disrupting the remainder of the gene (58). A proposed mechanism of how ITD helps blast cells is by conferring a growth advantage and self-renewal capacity (59). In a large retrospective review of AML patients in Japan, 43 subjects were identified as having Flt3-ITD and had significantly worse 5-year DFS of <20%, compared to those without Flt3-ITD (5-year DFS of 50%) (57). Additionally, multiple CCG studies, 2891, 2941, and 2961, have reviewed pediatric patients and identified that Flt3 ITDs were an independent poor prognostic factor and that children with an ITD allelic ratio >0.4 had poor outcomes as compared to those with Flt3-wild type (60, 61). In the most recent COG study, Flt3 has been added for risk stratification and those with high allelic ratio of Flt3–ITD will receive more intensive chemotherapy with a Flt3 inhibitor, and will receive a HSCT in first remission (62). As discussed above, Flt3 inhibitors are currently being studied in pediatric phase III trials, in infant ALL (lestaurtinib) and *de novo* AML with Flt3–ITD (sorafenib). Continued improvement in outcomes in high-risk AML will require continued research for novel therapeutic agents.

## Adoptive Cellular Therapy for Pediatric Acute Lymphoblastic Leukemia

Immunotherapy has emerged as a promising treatment for patients with relapsed-refractory acute leukemia over the past decade. In the simplest sense, the immune system is responsible for surveying the body and eliminating foreign substances that can cause harm, including the clearance of cells that have acquired malignant potential. Conceptually, Schreiber et al. has described cancer immunosurveillance and cancer progression as a cell editing process proceeding through three sequential phases; elimination, equilibrium, and escape. If the innate and adaptive immune systems are unable to rid the body of highly immunogenic transformed cells (elimination phase), the tumor variant cells become dormant and undergo additional genetic changes in the face of immune pressure (equilibrium phase) (63). If immunosurveillance fails to recognize and destroy edited tumor cells, tumor evasion mechanisms can be acquired such as loss of tumor-associated antigens (TAAs) or down-regulation of major histocompatibility complex (MHC) antigens (63, 64). This creates a state of immune tolerance which in turn creates a microenvironment that allows the tumor to flourish (escape phase) (63). Genetically engineered T cells using a recombinant T cell receptor (TCR) have been implemented in an attempt to re-direct the immune system. This recombinant T cell gains high specificity for antigens expressed on tumor cells (65). These engineered TCRs showed promise in early clinical trials (64), however had multiple limitations; namely, human leukocyte antigen (HLA) restriction that limits patient eligibility, dependency on MHC expression by tumor cells, and toxicity (65).

More recently, adoptive cellular therapy has now included T cells genetically modified using CARs that are independent of MHC restriction. The original concept of CARs was first introduced by Gross et al. in 1989, and significant work has been done since then to create more sophisticated domains that can enhance activation, expansion, and survival of modified T cells (66, 67). CARs are customized receptors that are composed of an extracellular antigen-binding domain targeting antigens expressed on the malignant cells (such as CD19 expressed on B cell malignancies) combined with the intracellular signaling domains of the T cell (64). The antigen-binding domain typically consists of single protein chains derived from monoclonal antibody fragment variable regions from heavy and light chains connected by a short linker sequence to make a singe chain (scFvs) (67, 68). First-generation CARs incorporated only the CD3-ζ chain of the TCR/CD complex as the signaling domain but failed to generate potent antitumor effects. To improve potency, T cell costimulatory domains have now been included generating second (and third) generation CARs that incorporate TCR-ζ plus a costimulatory signal such as the CD28 endodomain. Indeed, a clinical trial in which patients with refractory non-Hodgkin’s lymphoma (NHL) were simultaneously infused equal amounts of first generation CD-19 targeted CAR T cells, or second-generation CD-19 CAR T cells clearly demonstrated that costimulation improved the *in vivo* expansion and persistence of CAR-modified T cells when combined with the costimulatory CD28 domain (69). A costimulatory endodomain has since been incorporated into most of CAR constructs to enhance the signaling strength, persistence, and overall potency of the CAR T cells (70). Multiple costimulatory domains are currently being investigated, CD28, 4-1BB, OX40, ICOS, and DAP10; however, no consensus has been reached regarding which costimulatory domain is superior.

## Antigen Selection in B ALL

Genetically engineered T cells have become a more realistic option for those patients with refractory or multiply relapsed acute leukemia for which conventional therapies have failed. Hematologic malignancies have been a prime example in the development of antibody-based immunotherapy (including CAR T cells which use an antibody-derived binding domain) because of cell surface antigens well known through flow cytometry; many of which have restricted expression to hematopoietic lineages with potential for regeneration of the non-malignant cells expressing the same target after completion of therapy. Ideally, an antigen target is one that has high expression on the malignant cell surface, and is not detected on normal tissues (or at least limited), therefore maximizing selectivity and minimizing off-tumor toxicity (71). In the context of cell therapy for leukemia, CD19 has been the most targeted antigen thus far with multiple trials centered on relapsed or refractory acute B cell leukemia using anti-CD19 antibodies. This antigen is solely expressed on B cell lineage, sparing other normal tissues, and is highly expressed on B ALL. Blinatumamab is a bispecific T cell engaging antibody (BiTE), anti-CD19/CD3, that has shown efficacy in adult and pediatric patients with relapsed or refractory ALL (72, 73). In a phase I/II pediatric study, 32% of pediatric patients with refractory ALL entered into a cytologic complete remission. Currently, a multi-center phase II/III trial (AALL1331) is underway to determine if using blinatumamab in upfront relapsed leukemia therapy increases leukemia-free survival in pediatric patients.

CD19 CAR therapy has revolutionized immunotherapy for hematologic malignancies with major success in multiple pediatric phase I studies (74, 75). In one of the largest pediatric studies to date, 30 patients were included, with 25 patients <22 years old. The CAR construct used was a second generation with a 41-BB costimulatory domain. Morphologic complete remission was induced in 27/30 patients, with 81% of those having MRD-negative disease. The 6-month EFS was 67% and OS was 78% (74). Lee et al. conducted a phase 1 study with CD19 second-generation CAR using a CD28 costimulatory domain in 21 patients and had a CR of 67%, with 12 patients (86%) achieving MRD-negative disease. OS was 56% for patients with a median of 10 months followup. Of those with MRD-negative disease, 10 of them went on to HSCT and all remained disease free at 10 months post HSCT (75). A novel mechanism of tumor escape has emerged with CAR and bispecific antibody therapy resulting in relapses with leukemia that no longer express the targeted CD19 epitope (74–76). The loss of antigen is a potential hurdle in the development of immunotherapy, thereby making it imperative to develop strategies to optimize, including the identification of additional targets.

CD22 is another common B-lineage marker that is expressed on leukemia and for which immunotherapeutic targeting has recently been developed. Two different CD22 immunotoxins have been tested against B ALL in clinical trials. In a phase 1 study, moxetumomab pasudotox (anti-CD22 pseudomonas exotoxin conjugate) showed activity in pediatric patients who had refractory disease. Twenty-three patients were enrolled in this phase 1 study and 7/23 patients either had a PR (with >50% blast reduction) or a CR (77, 78). Another CD22 immunotoxin (inotuzomab) has been tested primarily in adults with ALL and has demonstrated overall response rates of 58% in relapsed and refractory adult patients with acute lymphocytic leukemia (79). Recently, a CD22 CAR has been developed and shown to have comparable activity in murine xenografts when compared to CD19 CAR (80). A phase I study is underway using a CD22 CAR in patients with relapsed/refractory acute leukemia.

In order to identify novel antigenic targets and to improve selectivity for malignancy, flow cytometric and genomic data have been analyzed. A bioinformatics approach using gene-expression databases (containing multiple tumor samples annotated often with additional clinical and laboratory information) compares expression profiles between normal tissues and tumors of interest and can identify potential targets with a favorable expression profile. For example, genomic profiling of ALL has demonstrated recurring abnormalities in the CRLF2 gene resulting in overexpression of the encoded protein TSLPR in approximately 5–10% of B ALL. Importantly, overexpression of CRLF2 seems to confer a risk of relapse. As a cell surface protein, this oncogenic protein can be targeted immunotherapeutically. In fact, a TSLPR CAR has demonstrated potent activity in preclinical models (81). The large set of genomic data generated by expression profiling and next generation sequencing can be further analyzed using algorithms that “filter” for predicted cell surface expression. This approach has been applied to a number of pediatric malignancies, including B cell ALL and T cell ALL (82, 83).

To combat common toxicity seen in CD 19 trials, a more specific targeted CAR has been created in order to try and minimize B cell aplasia. Exploiting the monoclonality of malignant B cells, a recent phase I study used clonal restriction to create a k-light chain CAR that specifically targets k+ leukemia and lymphomas. Ten patients have been treated to date, with three dose levels assessed. Results of five NHL patients have showed promise with two patients entering a CR and one other showing partial response. This preliminary study has shown that CAR therapy restricted to k-light chain has been effective in patients with k+ lymphoma with diminished effects on the non-malignant B cell population (84).

## Immunotherapeutic Targets in Pediatric T Cell ALL

As reported in Orentas et al. 2014, Gene Expression Omnibus (GEO) data containing over 100 samples of T cell ALL were analyzed to determine which genes encoding cell surface proteins were most overexpressed in pediatric T cell leukemia in order to identify novel antigens that may be amenable to targeted therapy. One of the top transcripts that was notably overexpressed in T cell ALL as compared to PBMCs was TALLA (83). TALLA has been previously described by Seon et al. in the 1980s as a hybridoma monoclonal antibody that had specific target selectivity for T cell leukemia. Hara and Seon showed that this hybridoma, when injected in mice with T cell leukemia, resulted in *in vivo* suppression of tumor growth without overt toxicity (85, 86). Using gene-expression data combined with known activity of a monoclonal antibody, TALLA may be an area of future research in relapsed or refractory T cell ALL but will require validation that this antigen is expressed on the surface of the majority of primary T cell ALL samples.

As discussed previously, ETP-ALL has typically been more difficult to treat, with more induction failure than patients with more mature T cell ALL. Recently, in preclinical studies, Maude et al. demonstrated aberrant JAK/STAT pathway in ETP-ALL patient samples. Patient-derived xenograft models were established and ruxolitinib, a JAK1/2 inhibitor, demonstrated robust activity as a single agent, with a statistically significant reduction in peripheral blasts as compared to controls in 5/6 xenograft models. Continued work is needed in this area to further establish targets for novel treatments (87).

Mamonkin et al. recently developed a CAR directed at CD5 for T cell ALL that showed promising *in vitro* and *in vivo* activity. They demonstrated that CD5 CAR T cells were cytotoxic to five different CD5+ T cell ALL and T cell lymphoma lines *in vitro*, and had no effect on CD5^−^ lines (88). Additionally, they were able to show that these CD5 CAR T cells had tumor suppression capabilities when co-cultured for longer periods of time with these CD5^+^ cell lines. They were also able to show *in vivo* activity of CD5 CAR T cells against T cell ALL in xenograft models, both when infused early and late after leukemia injection was given. Xenografts treated with experimental CD5 CAR T cells had statistically significant survival advantage as compared to control groups given CD 19 CAR (88). However, complete eradication was not seen in these models and leukemia blasts retained CD5 cell surface expression, suggesting lack of persistence of CAR. One of the major challenges of producing a CAR T cell directed at an antigen on most naïve T cells is protecting healthy T cells from destruction by CAR therapy. These authors concluded that self-killing of T cells was limited; T cells co-cultured with CD5 CAR initially declined in number, however had comparable sustained expansion when compared to the control CD19 CAR used (88). These data are promising in light of new antigens being discovered specifically for a disease subtype in pediatrics that has limited options in targeted therapy.

## CARS in Pediatric AML

As stated above, pediatric patients with relapsed AML have limited therapeutic options with HSCT providing the only curative potential. Inducing a remission in order to get to transplant in these relapsed patients becomes challenging due to chemorefractory disease. CD33 targeting has been an area of investigation in pediatric AML, as seen with the anti-CD33 monoclonal antibody, Gemtuzumab ozogamicin (GO). In a pediatric clinical trial, it appeared that children receiving GO had a statistically significantly improved 3-year EFS, 53 vs. 47% (*P* = 0.05), however the overall survival did not differ in the groups, thus it was unclear if GO had any added benefit (89). Several studies are ongoing testing the efficacy of CD33-directed CAR therapy. Kenderian et al. developed four constructs using the scFv region of GO and tested these targeted agents against AML cell lines, patient AML samples, and xenografts. The CD33 CARs demonstrated significant cytotoxicity and cellular degranulation against AML cell lines, with significant *in vitro* activity against primary AML samples as well. Significant reduction in tumor burden and a survival advantage was seen in mice injected with CD33 CAR vs. those injected with control T cells (90). Toxicity associated with this CAR resulted in decrease in myeloid progenitor cells, which was expected based on CD33 expression (90). Similarly, O’Hear et al. recently described a novel second-generation CD33 CAR that incorporated 41-BB costimulatory endodomain used in preclinical models. AML cell lines, xenografts, and primary pediatric samples were assessed. Specific tumor killing of CD33^+^ AML cell lines was seen when co-cultured with CD33 CAR, and these cultures had higher levels of cytokines when compared to the cell lines co-cultured with control vector transduced T cells. This same result was seen with primary AML samples, and interestingly, the extent of killing did not correlate with the cell surface level of expression of CD33 (91). Additionally, xenografts were established and studied in two ways: (1) to determine if CAR T cells could prevent the development of AML and (2) to determine if CAR T cells could treat AML. Mice were injected with AML and, in the first model, were immediately injected with either CAR T cells, viral transduced T cells, or saline. Results showed rapid development of leukemia in the two control groups with <1% of AML cells detected in those injected with CAR T cells. Also in established leukemia models, treated as per the three groups above, CAR T cells resulted in significant reduction in tumor burden with a statistically significant survival advantage compared to mice treated with control cells (91). Results of these preclinical models are promising for creating a CAR construct to combat relapsed or refractory AML.

In an attempt to overcome the significant hematopoietic toxicity associated with CD33 targeted therapy, Kenderian et al. developed a transient CAR using electroporation of T cells with RNA-modified CD33 CAR. Results of this preclinical model showed similar eradication of leukemia cells as compared to lentiviral transduced CD33 CAR, albeit transient *in vitro* activity. It was then combined with chemotherapy *in vivo* and shown to have cytotoxic effects with enhanced CAR persistence (90). This safety mechanism provided by transient persistence is important as it could avoid prolonged myelosuppression in AML patients.

Multiple groups have recently reported preclinical activity against CD123 in AML. Pizzitola et al. recently described activity with cytokine-induced killer cells (CIK) that had been genetically modified to express CD33-CAR and CD123-CAR. What they found was that mice treated with CIK with either CD33 or CD123 bound CAR had significant reduction in tumor burden than those in the control group. Interestingly, when leukemia returned, analysis was performed and residual AML cells continued to express CD33/123, thus concluding that these cells did not develop the resistance mechanisms employed as previously described in CD19 CAR immunotherapy (92). Also, Tettamanti et al. recently described CD123 as a potent AML target due to its high expression on AML cells and AML leukemia stem cells (AML-LSCs) and low levels of expression on hematopoietic stem cells. *In vitro* studies demonstrated CIK with anti-CD33 or anti-CD123 had equal AML cytotoxic effects; however, CD123-CAR had a safer toxicity profile toward progenitor cells (93). Similarly, Mardiros et al. developed a CD123-CAR that showed specific activity against primary AML samples, in addition to *in vivo* activity in xenograft AML models. This study showed delayed leukemia relapse in xenografts treated with CAR therapy vs. mock T cells; however, the treatment group did not have any long-term survivors (94) Gill et al. recently demonstrated CD123-CAR activity in primary samples and xenograft models, and was able to show long-term survivors after treatment with CD123-CARs. This study differed from the others in that they saw significant hematopoietic progenitor toxicity with their CD123-CAR, recommending usage as a bridge or conditioning regimen for HSCT (95). Finally, CAR T cells targeting the Lewis Y antigen and the folate receptor β have also shown activity against AML (96, 97). Thus, there are multiple potential targets for CAR T cells in AML. However, it remains to be seen whether clinical activity will be as good as that seen with CD19 CAR T cells in ALL and the extent to which toxicity against non-malignant myeloid cells limit development.

## The Potential of Combinatorial CAR Targeting in Pediatric Leukemia

**Figure 1:**
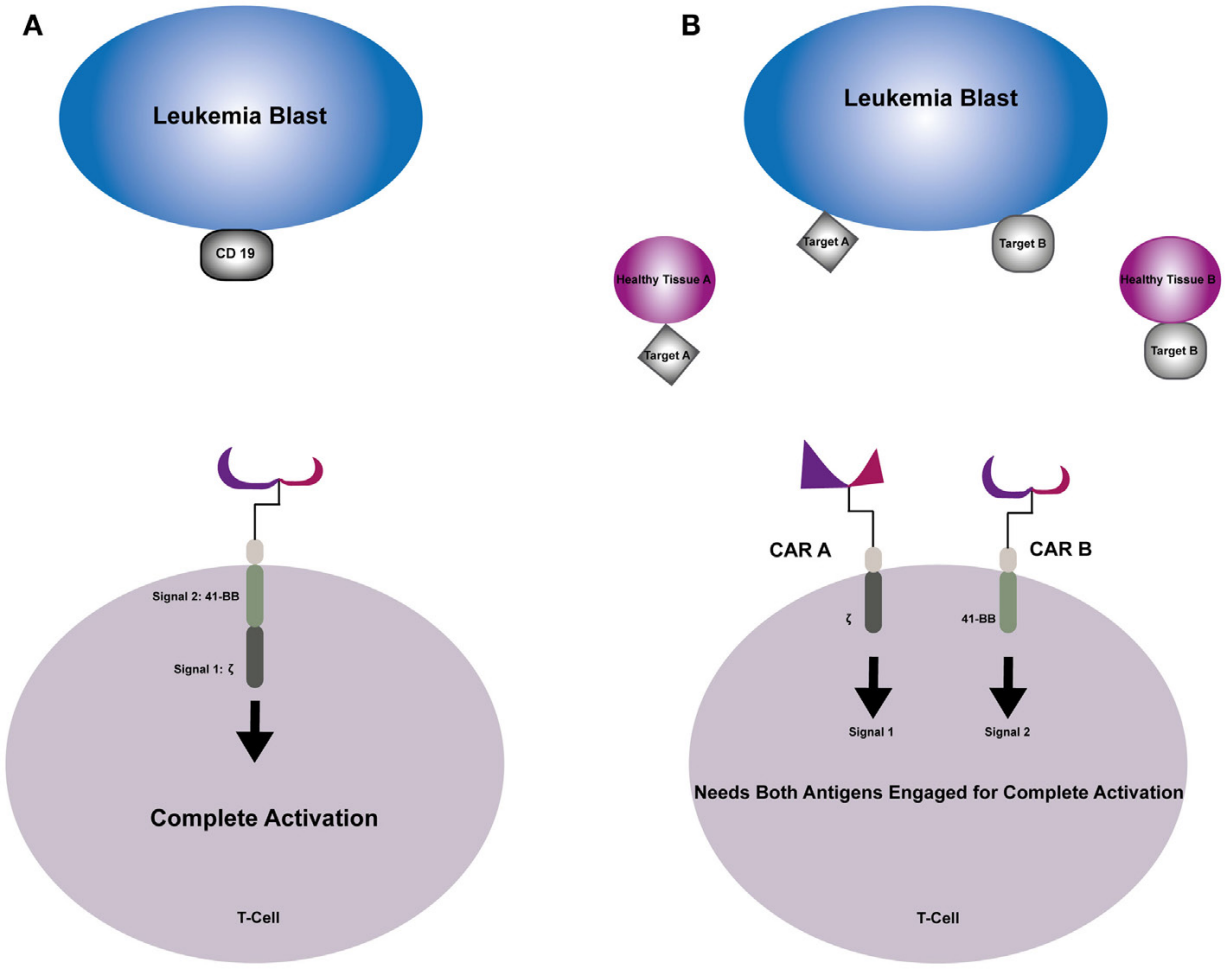
**(A)** Second-generation CAR engages the selected target antigen and produces complete T-cell activation through signal 1 provided by the TCR–zeta and signal 2 provided by costimulatory endodomain 41-BB. **(B)** Combinatorial CAR T-cell that has two antigens; CAR A provides signal 1 when engaged with Target A and CAR B provides signal 2 when engaged with Target B. Neither signal can trigger complete T-cell activation individually, sparing healthy tissue that express one of the antigens.

Due to its universal expression on B cell malignancies, CD19 has been an attractive target for CAR-directed therapies. Its success hinges on the fact that although CD19 is expressed on normal B cells, the toxicity associated with aplasia of this cellular compartment is tolerable as seen in recent trials (74, 75). Targeting of other acute hematologic malignancies, AML and T cell ALL, has been more challenging. Targeting cell surface molecules universal to these cell types is often detrimental to normal myeloid and T cell compartments, making it extremely difficult to identify highly specific CAR targets. In order to potentiate antitumor activity, and reduce off-tumor toxicity of normal cellular compartments, combinatorial approaches are being developed (Figure 1). This proof of concept was introduced by Grada et al. as a tandem CAR using CD19 and HER2 as antigens. This construct was shown to have activity against cell lines that had single antigens in addition to having synergistic effect when both antigens were simultaneously encountered (98). Kloss et al. used a combinatorial approach transducing T cells with both a CAR that provides suboptimal activation upon binding of one antigen, and a chimeric costimulatory receptor that binds a second antigen. Using T cells that were co-transduced with two prostate antigens, tumors expressing both antigens were effectively targeted and destroyed while sparing tumors that expressed one sole antigen (99). Lanitis et al. identified two potential antigen targets for CAR development for use in epithelial ovarian cancers using mesothelin (meso) and a-folate receptor (Fra). Ovarian tumors generally overexpress both these antigens, while coexpression on normal tissues is rare. Two separate CARs were created, one with T cell activation signal 1 (meso-CD3ζ) and the other with costimulatory signal 2 (Fra-CD28), and T cells where then transduced to co-express both CARs. By using this physical separation barrier, only cells expressing both antigens would bind and elicit complete T cell activation needed for antitumor activity. This preclinical model demonstrated potent anticancer activity *in vitro* and *in vivo* that was superior to first-generation CARs and comparable to standard second-generation CARs. The major advantage of this new combinatorial CAR over a standard second-generation CAR was dissociated signaling of two antigens yielded a better side effect profile against cross-reactivity with normal healthy tissues expressing a single antigen (100).

Combinatorial targeting approaches may be particularly well suited for pediatric acute leukemia, specifically AML and T cell ALL that express multiple hematopoietic antigens. Additionally, varying reports (10–30%) of aberrant lymphoid or myeloid-associated markers have been demonstrated in pediatric studies of patients with acute leukemia. Conflicting reports as to the prognostic implications of aberrant phenotypes have been shown in prospective pediatric studies performed in the 1990s (101–103). Pui et al. found that 16% of pediatric patients consecutively enrolled on clinical trials had aberrant expression of myeloid antigens on their ALL blasts, however found that it did not have an effect on remission or EFS (101). On the other hand, Wiersma et al. found that 22% of newly diagnosed pediatric patients expressed myeloid antigens on their leukemia blasts and in a multivariate analysis, it was found to be the most significant poor prognostic predictor of relapse in childhood ALL (102). Although the prognostic significance remains controversial, the presence of aberrant myeloid antigens is a common occurrence in childhood ALL (104, 105). Using this to our advantage, one could construct a combinatorial CAR that has more specificity for these leukemia blasts and spare normal HSCs.

## Other Approaches to Improve Safety

Careful selection of TAAs is of paramount importance due to the potency of genetically modified T cells and the potential on-target, off-tumor toxicity that has been reported. One patient treated for metastatic colon cancer with Her2/neu CAR T cells died 5 days later from respiratory failure attributable to cytokine storm potentially due to low levels of Her2/neu found in lung epithelium (106). This highlights the importance of assessing for cross-reactivity on normal cells during the preclinical development of genetically engineered cells. However, it is likely that it will be challenging to identify antigens that are exclusively expressed on tumor cells or that are expressed only by normal cells for which off-tumor toxicity will be tolerated (such as CD19 on B cells). Indeed, patients responding to CD19-CAR therapy develop B cell aplasia noted that can be managed by immunoglobulin infusions. Thus, approaches to control expansion or persistence of genetically engineered cells will be important for the clinical development. For example, inducible caspase 9, an intrinsic apoptotic pathway, can be incorporated into CAR T cell as a “suicide gene.” Following an allogeneic transplant, patients were given a donor lymphocyte infusion of cells modified to express the inducible caspase 9 gene, and had more than 90% of T cells eliminated in 30 min when given 1 dose of a dimerizing drug (107). This construct is currently being incorporated into CAR T cells in the context of clinical trials. Additionally, the use of RNA electroporated CAR constructs allows for transient expression of CAR in T cells with retention of potent activity provides a possible strategy to improve the safety of CAR T cell therapy (90, 108) Finally, using CAR T cells that express a truncated EGFR can be used as a safety feature for hematologic CAR targeted therapy. Human epidermal growth factor receptor is not expressed on cells of the hematopoietic and lymphopoietic systems; therefore, it will not have an impact on progenitor cells. Using truncated EGFR-CAR is helpful as it provides a cell surface marker for *in vivo* tracking of adoptive T cell therapy. When therapy is completed or toxicities have been noted, administration of a monoclonal antibody, cetuximab, results in *in vivo* cell ablation, thereby employing a safety mechanism that can ameliorate potential long-term CAR side effects (109).

A relatively common and potentially life-threatening toxicity seen in CD19 CAR trials is cytokine-release syndrome (CRS), a systemic, immune-mediated inflammatory response. CRS ranges in severity from mild (fevers and myalgia) to life threatening (capillary leak, respiratory failure, hypotension) and is often correlated with high peak levels of IL-6, C-reactive protein, ferritin, and IL-2 (*P* < 0.05) (74, 75). It was also noted that higher patient blast percentages prior to therapy correlated with more severe CRS and that CRS is often associated with clinical response (74). For those with more severe CRS, IL-6 blockade and/or glucocorticoids can mediate rapid improvement (74, 75). Thus, there is interest in developing standard approaches for intervention. As suggested by Lee et al., creating an algorithm where IL-6 blockade is used as the first line therapy for treatment of severe CRS can systematically guide therapy for CRS perhaps avoiding mortality (75).

## Conclusion

Exceptional progress has been made over the last two decades with genetically modified T cells with recent dramatic success using CAR T cells for pediatric leukemia. With continued identification of targets, the possibilities of CAR therapy continue to expand. Careful preclinical and clinical development will be required for the safe and successful incorporation of genetically modified T cells into treatment paradigms for cancer. Almost certainly, approaches to manage potential toxicity to non-malignant tissues due to on-target, off-tumor reactivity will be required. One strategy is the development of combinatorial therapy using T cells expressing multiple CAR constructs thus controlling reactivity based on expression of combinations of targets. Pediatric leukemia may be particularly amenable to such approaches due to aberrant expression of cell surface molecules not typically found on normal hematopoietic cells.

## Conflict of Interest Statement

The content of this publication does not necessarily reflect the views of policies of the Department of Health and Human Services, nor does mention of trade names, commercial products, or organizations imply endorsement by the U.S. Government. The authors declare that the research was conducted in the absence of any commercial or financial relationships that could be construed as a potential conflict of interest.

## References

[1] Howlader N Noone A Krapcho M Garshell J Miller D Altekruse S SEER Cancer Statistics Review, 1975–2011. Bethesda, MD: National Cancer Institute (2014).

[2] Hunger SP Lu X Devidas M Camitta BM Gaynon PS Winick NJ Improved survival for children and adolescents with acute lymphoblastic leukemia between 1990 and 2005: a report from the Children’s Oncology Group. J Clin Oncol (2012) 30:1663–9. 10.1200/JCO.2011.37.8018 22412151 PMC3383113

[3] Rabin Z-MP Classification of Newly Diagnosed Acute Lymphoblastic Leukemia (ALL) Children’s Oncology Group Protocol: AALL08B1 (2015).

[4] Schultz KR Pullen DJ Sather HN Shuster JJ Devidas M Borowitz MJ Risk- and response-based classification of childhood B-precursor acute lymphoblastic leukemia: a combined analysis of prognostic markers from the Pediatric Oncology Group (POG) and Children’s Cancer Group (CCG). Blood (2007) 109:926–35. 10.1182/blood-2006-01-024729 17003380 PMC1785141

[5] Carroll W Hunger S Borowitz M Bhojwani D Willman C Devidas M Risk-adapted therapy for children with acute lymphoblastic leukemia (ALL): the Children’s Oncology Group (COG) approach. Ann Hematol (2008) 87:S42–4. 10.1007/s00277-008-0443-6

[6] Henze G v Stackelberg A Eckert C. ALL-REZ BFM – the consecutive trials for children with relapsed acute lymphoblastic leukemia. Klin Padiatr (2013) 225(Suppl 1):S73–8. 10.1055/s-0033-1337967 23700062

[7] Tallen G Ratei R Mann G Kaspers G Niggli F Karachunsky A Long-term outcome in children with relapsed acute lymphoblastic leukemia after time-point and site-of-relapse stratification and intensified short-course multidrug chemotherapy: results of trial ALL-REZ BFM 90. J Clin Oncol (2010) 28:2339–47. 10.1200/JCO.2009.25.1983 20385996

[8] Henze G Fengler R Hartmann R Kornhuber B Janka-Schaub G Niethammer D Six-year experience with a comprehensive approach to the treatment of recurrent childhood acute lymphoblastic leukemia (ALL-REZ BFM 85). A relapse study of the BFM group. Blood (1991) 78:1166–72. 1878583

[9] Gaynon PS Harris RE Altman AJ Bostrom BC Breneman JC Hawks R Bone marrow transplantation versus prolonged intensive chemotherapy for children with acute lymphoblastic leukemia and an initial bone marrow relapse within 12 months of the completion of primary therapy: Children’s Oncology Group study CCG-1941. J Clin Oncol (2006) 24:3150–6. 10.1200/JCO.2005.04.5856 16717292

[10] Bhojwani D Howard SC Pui C-H. High-risk childhood acute lymphoblastic leukemia. Clin Lymphoma Myeloma (2009) 9:S222. 10.3816/CLM.2009.s.016 19778845 PMC2814411

[11] Ritchey AK Pollock BH Lauer SJ Andejeski Y Barredo J Buchanan GR. Improved survival of children with isolated CNS relapse of acute lymphoblastic leukemia: a Pediatric Oncology Group study. J Clin Oncol (1999) 17:3745–52. 10577846 10.1200/JCO.1999.17.12.3745

[12] Barredo JC Devidas M Lauer SJ Billett A Marymont M Pullen J Isolated CNS relapse of acute lymphoblastic leukemia treated with intensive systemic chemotherapy and delayed CNS radiation: a Pediatric Oncology Group study. J Clin Oncol (2006) 24:3142–9. 10.1200/JCO.2005.03.3373 16809737

[13] Raetz EA Borowitz MJ Devidas M Linda SB Hunger SP Winick NJ Reinduction platform for children with first marrow relapse of acute lymphoblastic leukemia: a Children’s Oncology Group study [corrected]. J Clin Oncol (2008) 26:3971–8. 10.1200/JCO.2008.16.1414 18711187 PMC2654313

[14] Freyer DR Devidas M La M Carroll WL Gaynon PS Hunger SP Postrelapse survival in childhood acute lymphoblastic leukemia is independent of initial treatment intensity: a report from the Children’s Oncology Group. Blood (2011) 117:3010–5. 10.1182/blood-2010-07-294678 21193696 PMC3062307

[15] Rivera GK Zhou Y Hancock ML Gajjar A Rubnitz J Ribeiro RC Bone marrow recurrence after initial intensive treatment for childhood acute lymphoblastic leukemia. Cancer (2005) 103:368–76. 10.1002/cncr.20743 15599932

[16] Schultz KR Carroll A Heerema NA Bowman WP Aledo A Slayton WB Long-term follow-up of imatinib in pediatric Philadelphia chromosome-positive acute lymphoblastic leukemia: Children’s Oncology Group study AALL0031. Leukemia (2014) 28:1467–71. 10.1038/leu.2014.30 24441288 PMC4282929

[17] Roberts KG Li Y Payne-Turner D Harvey RC Yang YL Pei D Targetable kinase-activating lesions in Ph-like acute lymphoblastic leukemia. N Engl J Med (2014) 371:1005–15. 10.1056/NEJMoa1403088 25207766 PMC4191900

[18] Den Boer ML van Slegtenhorst M De Menezes RX Cheok MH Buijs-Gladdines JG Peters ST A subtype of childhood acute lymphoblastic leukaemia with poor treatment outcome: a genome-wide classification study. Lancet Oncol (2009) 10:125–34. 10.1016/S1470-2045(08)70339-5 19138562 PMC2707020

[19] Raimondi SC Zhou Y Mathew S Shurtleff SA Sandlund JT Rivera GK Reassessment of the prognostic significance of hypodiploidy in pediatric patients with acute lymphoblastic leukemia. Cancer (2003) 98:2715–22. 10.1002/cncr.11841 14669294

[20] Nachman JB Heerema NA Sather H Camitta B Forestier E Harrison CJ Outcome of treatment in children with hypodiploid acute lymphoblastic leukemia. Blood (2007) 110:1112–5. 10.1182/blood-2006-07-038299 17473063 PMC1939895

[21] Mehta PA Zhang MJ Eapen M He W Seber A Gibson B Transplantation outcomes for children with hypodiploid acute lymphoblastic leukemia. Biology of blood and marrow transplantation. J Am Society Blood Marrow Transplant (2015) 21:1273–7. 10.1016/j.bbmt.2015.04.008 25865650 PMC4465998

[22] Coustan-Smith E Sancho J Hancock ML Boyett JM Behm FG Raimondi SC Clinical importance of minimal residual disease in childhood acute lymphoblastic leukemia. Blood (2000) 96:2691–6. 11023499

[23] Borowitz MJ Devidas M Hunger SP Bowman WP Carroll AJ Carroll WL Clinical significance of minimal residual disease in childhood acute lymphoblastic leukemia and its relationship to other prognostic factors: a Children’s Oncology Group study. Blood (2008) 111:5477–85. 10.1182/blood-2008-01-132837 18388178 PMC2424148

[24] Borowitz MJ Wood BL Devidas M Loh ML Raetz EA Salzer WL Prognostic significance of minimal residual disease in high risk B-ALL: a report from Children’s Oncology Group study AALL0232. Blood (2015) 126:964–71. 10.1182/blood-2015-03-633685 26124497 PMC4543229

[25] Sramkova L Muzikova K Fronkova E Krejci O Sedlacek P Formankova R Detectable minimal residual disease before allogeneic hematopoietic stem cell transplantation predicts extremely poor prognosis in children with acute lymphoblastic leukemia. Pediatr Blood Cancer (2007) 48:93–100. 10.1002/pbc.20794 16521130

[26] Leung W Pui CH Coustan-Smith E Yang J Pei D Gan K Detectable minimal residual disease before hematopoietic cell transplantation is prognostic but does not preclude cure for children with very-high-risk leukemia. Blood (2012) 120:468–72. 10.1182/blood-2012-02-409813 22517895 PMC3398757

[27] Pulsipher MA Langholz B Wall DA Schultz KR Bunin N Carroll WL The addition of sirolimus to tacrolimus/methotrexate GVHD prophylaxis in children with ALL: a phase 3 Children’s Oncology Group/Pediatric Blood and Marrow Transplant Consortium trial. Blood (2014) 123:2017–25. 10.1182/blood-2013-10-534297 24497539 PMC3968388

[28] Pulsipher MA Carlson C Langholz B Wall DA Schultz KR Bunin N IgH-V(D)J NGS-MRD measurement pre- and early post-allotransplant defines very low- and very high-risk ALL patients. Blood (2015) 125:3501–8. 10.1182/blood-2014-12-615757 25862561 PMC4447864

[29] Leung W Campana D Yang J Pei D Coustan-Smith E Gan K High success rate of hematopoietic cell transplantation regardless of donor source in children with very high-risk leukemia. Blood (2011) 118:223–30. 10.1182/blood-2011-01-333070 21613256 PMC3138677

[30] Druley TE Hayashi R Mansur DB Zhang QJ Barnes Y Trinkaus K Early outcomes after allogeneic hematopoietic SCT in pediatric patients with hematologic malignancies following single fraction TBI. Bone Marrow Transplant (2009) 43:307–14. 10.1038/bmt.2008.327 19011666 PMC2792985

[31] Shi-Xia X Xian-Hua T Hai-Qin X Bo F Xiang-Feng T. Total body irradiation plus cyclophosphamide versus busulphan with cyclophosphamide as conditioning regimen for patients with leukemia undergoing allogeneic stem cell transplantation: a meta-analysis. Leuk Lymphoma (2010) 51:50–60. 10.3109/10428190903419130 20055658

[32] Oliansky DM Camitta B Gaynon P Nieder ML Parsons SK Pulsipher MA Role of cytotoxic therapy with hematopoietic stem cell transplantation in the treatment of pediatric acute lymphoblastic leukemia: update of the 2005 evidence-based review. Biol Blood Marrow Transplant (2012) 18:505–22. 10.1016/j.bbmt.2011.12.585 22209888

[33] Balduzzi A Valsecchi MG Uderzo C De Lorenzo P Klingebiel T Peters C Chemotherapy versus allogeneic transplantation for very-high-risk childhood acute lymphoblastic leukaemia in first complete remission: comparison by genetic randomisation in an international prospective study. Lancet (2005) 366:635–42. 10.1016/S0140-6736(05)66998-X 16112299

[34] Conter V Valsecchi MG Parasole R Putti MC Locatelli F Barisone E Childhood high-risk acute lymphoblastic leukemia in first remission: results after chemotherapy or transplant from the AIEOP ALL 2000 study. Blood (2014) 123:1470–8. 10.1182/blood-2013-10-532598 24415536

[35] Pinkel D ‘Allogeneic marrow transplantation in children with acute leukemia: a practice whose time has gone’: twenty years later. Leukemia (2009) 23:2189–96. 10.1038/leu.2009.132 20016481

[36] Pulsipher MA Hunger SP Gamis AS Wall DA Grupp SA Allogeneic marrow transplantation in children with acute leukemia: careful comparison with chemotherapy alternatives required. Leukemia (2010) 24:1212–6. 10.1038/leu.2010.72 20428198

[37] Kotecha RS Gottardo NG Kees UR Cole CH. The evolution of clinical trials for infant acute lymphoblastic leukemia. Blood Cancer J (2014) 4:e200. 10.1038/bcj.2014.17 24727996 PMC4003413

[38] Hilden JM Dinndorf PA Meerbaum SO Sather H Villaluna D Heerema NA Analysis of prognostic factors of acute lymphoblastic leukemia in infants: report on CCG 1953 from the Children’s Oncology Group. Blood (2006) 108:441–51. 10.1182/blood-2005-07-3011 16556894 PMC1895499

[39] Reaman GH Sposto R Sensel MG Lange BJ Feusner JH Heerema NA Treatment outcome and prognostic factors for infants with acute lymphoblastic leukemia treated on two consecutive trials of the Children’s Cancer Group. J Clin Oncol (1999) 17:445–55. 10080584 10.1200/JCO.1999.17.2.445

[40] Pieters R Schrappe M De Lorenzo P Hann I De Rossi G Felice M A treatment protocol for infants younger than 1 year with acute lymphoblastic leukaemia (Interfant-99): an observational study and a multicentre randomised trial. Lancet (2007) 370:240–50. 10.1016/S0140-6736(07)61126-X 17658395

[41] Isoyama K Eguchi M Hibi S Kinukawa N Ohkawa H Kawasaki H Risk-directed treatment of infant acute lymphoblastic leukaemia based on early assessment of MLL gene status: results of the Japan Infant Leukaemia Study (MLL96). Br J Haematol (2002) 118:999–1010. 10.1046/j.1365-2141.2002.03754.x 12199778

[42] Tomizawa D Koh K Hirayama M Miyamura T Hatanaka M Saikawa Y Outcome of recurrent or refractory acute lymphoblastic leukemia in infants with MLL gene rearrangements: a report from the Japan Infant Leukemia Study Group. Pediatr Blood Cancer (2009) 52:808–13. 10.1002/pbc.21975 19229974

[43] Nagayama J Tomizawa D Koh K Nagatoshi Y Hotta N Kishimoto T Infants with acute lymphoblastic leukemia and a germline MLL gene are highly curable with use of chemotherapy alone: results from the Japan Infant Leukemia Study Group. Blood (2006) 107:4663–5. 10.1182/blood-2005-11-4728 16478880

[44] Tomizawa D Koh K Sato T Kinukawa N Morimoto A Isoyama K Outcome of risk-based therapy for infant acute lymphoblastic leukemia with or without an MLL gene rearrangement, with emphasis on late effects: a final report of two consecutive studies, MLL96 and MLL98, of the Japan Infant Leukemia Study Group. Leukemia (2007) 21:2258–63. 10.1038/sj.leu.2404903 17690691

[45] Pui CH Gaynon PS Boyett JM Chessells JM Baruchel A Kamps W Outcome of treatment in childhood acute lymphoblastic leukaemia with rearrangements of the 11q23 chromosomal region. Lancet (2002) 359:1909–15. 10.1016/S0140-6736(02)08782-2 12057554

[46] Kang H Wilson CS Harvey RC Chen IM Murphy MH Atlas SR Gene expression profiles predictive of outcome and age in infant acute lymphoblastic leukemia: a Children’s Oncology Group study. Blood (2012) 119:1872–81. 10.1182/blood-2011-10-382861 22210879 PMC3293641

[47] Hilden BP A Phase III Study of Risk Directed Therapy for Infants with Acute Lymphoblastic Leukemia (ALL): Randomization of Highest Risk Infants to Intensive Chemotherapy ± FLT3 Inhibition Children’s Oncology Group: AALL0631. (2013).

[48] Brown P. Treatment of infant leukemias: challenge and promise. Hematology Am Soc Hematol Educ Program (2013) 2013:596–600. 10.1182/asheducation-2013.1.596 24319237 PMC4729208

[49] Seibel NL Steinherz PG Sather HN Nachman JB Delaat C Ettinger LJ Early postinduction intensification therapy improves survival for children and adolescents with high-risk acute lymphoblastic leukemia: a report from the Children’s Oncology Group. Blood (2008) 111:2548–55. 10.1182/blood-2007-02-070342 18039957 PMC2254538

[50] Einsiedel HG von Stackelberg A Hartmann R Fengler R Schrappe M Janka-Schaub G Long-term outcome in children with relapsed ALL by risk-stratified salvage therapy: results of trial acute lymphoblastic leukemia-relapse study of the Berlin-Frankfurt-Munster Group 87. J Clin Oncol (2005) 23:7942–50. 10.1200/JCO.2005.01.1031 16258094

[51] Coustan-Smith E Mullighan CG Onciu M Behm FG Raimondi SC Pei D Early T-cell precursor leukaemia: a subtype of very high-risk acute lymphoblastic leukaemia. Lancet Oncol (2009) 10:147–56. 10.1016/S1470-2045(08)70314-0 19147408 PMC2840241

[52] Wood BL Winter SS Dunsmore KP Devidas M Chen S Asselin B T-Lymphoblastic Leukemia (T-ALL) Shows Excellent Outcome, Lack of Significance of the Early Thymic Precursor (ETP) Immunophenotype, and Validation of the Prognostic Value of End-Induction Minimal Residual Disease (MRD) in Children’s Oncology Group (COG) Study AALL0434, 56th ASH Annual Meeting and Exposition. (2014).

[53] Dunsmore KP T-CELL PILOT PROTOCOL The Use of Modified BFM ± Compound 506U78 (Nelarabine) (NSC# 686673, IND #52611) in an Intensive Chemotherapy Regimen for the Treatment of T-Cell Leukemia Children’s Oncology Group: AALL00P2. (2005).

[54] Berg SL Blaney SM Devidas M Lampkin TA Murgo A Bernstein M Phase II study of nelarabine (compound 506U78) in children and young adults with refractory T-cell malignancies: a report from the Children’s Oncology Group. J Clin Oncol (2005) 23:3376–82. 10.1200/JCO.2005.03.426 15908649

[55] Kaspers GJ Creutzig U Pediatric acute myeloid leukemia: international progress and future directions. Leukemia (2005) 19:2025–9. 10.1038/sj.leu.2403958 16304569

[56] Wheatley K Burnett AK Goldstone AH Gray RG Hann IM Harrison CJ A simple, robust, validated and highly predictive index for the determination of risk-directed therapy in acute myeloid leukaemia derived from the MRC AML 10 trial. United Kingdom medical research council’s adult and childhood leukaemia working parties. Br J Haematol (1999) 107:69–79. 10.1046/j.1365-2141.1999.01684.x 10520026

[57] Kiyoi H Naoe T Nakano Y Yokota S Minami S Miyawaki S Prognostic implication of FLT3 and N-RAS gene mutations in acute myeloid leukemia. Blood (1999) 93:3074–80. 10216104

[58] Annesley CE Brown P. The biology and targeting of FLT3 in pediatric leukemia. Front Oncol (2014) 4:263. 10.3389/fonc.2014.00263 25295230 PMC4172015

[59] Kiyoi H Towatari M Yokota S Hamaguchi M Ohno R Saito H Internal tandem duplication of the FLT3 gene is a novel modality of elongation mutation which causes constitutive activation of the product. Leukemia (1998) 12:1333–7. 10.1038/sj.leu.2401130 9737679

[60] Smith FO Alonzo TA Gerbing RB Woods WG Arceci RJ Children’s Cancer G. Long-term results of children with acute myeloid leukemia: a report of three consecutive Phase III trials by the Children’s Cancer Group: CCG 251, CCG 213 and CCG 2891. Leukemia (2005) 19:2054–62. 10.1038/sj.leu.2403925 16136168

[61] Lange BJ Smith FO Feusner J Barnard DR Dinndorf P Feig S Outcomes in CCG-2961, a children’s oncology group phase 3 trial for untreated pediatric acute myeloid leukemia: a report from the Children’s Oncology Group. Blood (2008) 111:1044–53. 10.1182/blood-2007-04-084293 18000167 PMC2214754

[62] Aplenc RSL Meshinchi S A Phase III Randomized Trial for Patients with de novo AML using Bortezomib and Sorafenib (IND#114480; NSC# 681239, NSC# 724772) for Patients with High Allelic Ratio FLT3/ITD Children’s Oncology Group: AAML1031. (2014).

[63] Schreiber RD Old LJ Smyth MJ. Cancer immunoediting: integrating immunity’s roles in cancer suppression and promotion. Science (2011) 331:1565–70. 10.1126/science.1203486 21436444

[64] Marr LA Gilham DE Campbell JD Fraser AR. Immunology in the clinic review series; focus on cancer: double trouble for tumours: bi-functional and redirected T cells as effective cancer immunotherapies. Clin Exp Immunol (2012) 167:216–25. 10.1111/j.1365-2249.2011.04517.x 22235997 PMC3278687

[65] Chmielewski M Hombach AA Abken H. Antigen-specific T-cell activation independently of the MHC: chimeric antigen receptor-redirected T cells. Front Immunol (2013) 4:371. 10.3389/fimmu.2013.00371 24273543 PMC3822734

[66] Gross G Waks T Eshhar Z. Expression of immunoglobulin-T-cell receptor chimeric molecules as functional receptors with antibody-type specificity. Proc Natl Acad Sci USA (1989) 86:10024–8. 10.1073/pnas.86.24.10024 2513569 PMC298636

[67] Fry TJ Mackall CL. T-cell adoptive immunotherapy for acute lymphoblastic leukemia. Hematology Am Soc Hematol Educ Program (2013) 2013:348–53. 10.1182/asheducation-2013.1.348 24319203 PMC7569491

[68] Kershaw MH Westwood JA Slaney CY Darcy PK. Clinical application of genetically modified T cells in cancer therapy. Clin Transl Immunology (2014) 3:e16. 10.1038/cti.2014.7 25505964 PMC4232070

[69] Savoldo B Ramos CA Liu E Mims MP Keating MJ Carrum G CD28 costimulation improves expansion and persistence of chimeric antigen receptor-modified T cells in lymphoma patients. J Clin Invest (2011) 121:1822–6. 10.1172/JCI46110 21540550 PMC3083795

[70] Han EQ Li XL Wang CR Li TF Han SY. Chimeric antigen receptor-engineered T cells for cancer immunotherapy: progress and challenges. J Hematol Oncol (2013) 6:47. 10.1186/1756-8722-6-47 23829929 PMC3706354

[71] Vedi A Ziegler DS. Antibody therapy for pediatric leukemia. Front Oncol (2014) 4:82. 10.3389/fonc.2014.00082 24795859 PMC4000992

[72] Topp MS Kufer P Gokbuget N Goebeler M Klinger M Neumann S Targeted therapy with the T-cell-engaging antibody blinatumomab of chemotherapy-refractory minimal residual disease in B-lineage acute lymphoblastic leukemia patients results in high response rate and prolonged leukemia-free survival. J Clin Oncol (2011) 29:2493–8. 10.1200/JCO.2010.32.7270 21576633

[73] Gore L Zugmaier G Handgretinger R Locatelli F Trippett TM Rheingold SR Cytological and molecular remissions with blinatumomab treatment in second or later bone marrow relapse in pediatric acute lymphoblastic (ALL). J Clin Oncol (2013) 31.

[74] Maude SL Frey N Shaw PA Aplenc R Barrett DM Bunin NJ Chimeric antigen receptor T cells for sustained remissions in leukemia. N Engl J Med (2014) 371:1507–17. 10.1056/NEJMoa1407222 25317870 PMC4267531

[75] Lee DW Kochenderfer JN Stetler-Stevenson M Cui YK Delbrook C Feldman SA T cells expressing CD19 chimeric antigen receptors for acute lymphoblastic leukaemia in children and young adults: a phase 1 dose-escalation trial. Lancet (2015) 385:517–28. 10.1016/S0140-6736(14)61403-3 25319501 PMC7065359

[76] Topp MS Gokbuget N Zugmaier G Degenhard E Goebeler ME Klinger M Long-term follow-up of hematologic relapse-free survival in a phase 2 study of blinatumomab in patients with MRD in B-lineage ALL. Blood (2012) 120:5185–7. 10.1182/blood-2012-07-441030 23024237

[77] Shah NN Bhojwani D Silverman LB Whitlock JA Richards K Stetler-Stevenson M A novel anti-Cd22 immunotoxin, moxetumomab pasudotox (Ha22, Cat-8015): activity in pediatric patients with relapsed acute lymphoblastic leukemia (All) after allogeneic hematopoietic stem cell transplantation (Sct). Biol Blood Marrow Transplant (2012) 18:S234–234. 10.1016/j.bbmt.2011.12.091

[78] Mussai F Campana D Bhojwani D Stetler-Stevenson M Steinberg SM Wayne AS Cytotoxicity of the anti-CD22 immunotoxin HA22 (CAT-8015) against paediatric acute lymphoblastic leukaemia. Br J Haematol (2010) 150:352–8. 10.1111/j.1365-2141.2010.08251.x 20528877 PMC7316383

[79] Kantarjian H Thomas D Jorgensen J Kebriaei P Jabbour E Rytting M Results of inotuzumab ozogamicin, a CD22 monoclonal antibody, in refractory and relapsed acute lymphocytic leukemia. Cancer (2013) 119:2728–36. 10.1002/cncr.28136 23633004 PMC3720844

[80] Haso W Lee DW Shah NN Stetler-Stevenson M Yuan CM Pastan IH Anti-CD22-chimeric antigen receptors targeting B-cell precursor acute lymphoblastic leukemia. Blood (2013) 121:1165–74. 10.1182/blood-2012-06-438002 23243285 PMC3575759

[81] Qin H Cho M Haso W Zhang L Tasian SK Oo HZ Eradication of B-ALL using chimeric antigen receptor-expressing T cells targeting the TSLPR oncoprotein. Blood (2015) 126:629–39. 10.1182/blood-2014-11-612903 26041741 PMC4520878

[82] Orentas RJ Yang JJ Wen X Wei JS Mackall CL Khan J. Identification of cell surface proteins as potential immunotherapy targets in 12 pediatric cancers. Front Oncol (2012) 2:194. 10.3389/fonc.2012.00194 23251904 PMC3523547

[83] Orentas RJ Nordlund J He J Sindiri S Mackall C Fry TJ Bioinformatic description of immunotherapy targets for pediatric T-cell leukemia and the impact of normal gene sets used for comparison. Front Oncol (2014) 4:134. 10.3389/fonc.2014.00134 24959420 PMC4050364

[84] Ramos CA Savoldo B Liu EL Gee AP Mei ZY Grilley B Clinical responses in patients infused with T lymphocytes redirected to target kappa-light immunoglobulin chain. Mol Ther (2013) 21:S114–114. 10.1016/j.bbmt.2013.12.009

[85] Seon BK Negoro S Barcos MP. Monoclonal antibody that defines a unique human T-cell leukemia antigen. Proc Natl Acad Sci USA (1983) 80:845–9. 10.1073/pnas.80.3.845 6600841 PMC393477

[86] Hara H Seon BK. Complete suppression of in vivo growth of human leukemia cells by specific immunotoxins: nude mouse models. Proc Natl Acad Sci USA (1987) 84:3390–4. 10.1073/pnas.84.10.3390 3494997 PMC304876

[87] Maude SL Dolai S Delgado-Martin C Vincent T Robbins A Selvanathan A Efficacy of JAK/STAT pathway inhibition in murine xenograft models of early T-cell precursor (ETP) acute lymphoblastic leukemia. Blood (2015) 125:1759–67. 10.1182/blood-2014-06-580480 25645356 PMC4357583

[88] Mamonkin M Rouce RH Tashiro H Brenner MK. A T cell-directed chimeric antigen receptor for the selective treatment of T cell malignancies. Blood (2015) 126:983–92. 10.1182/blood-2015-02-629527 26056165 PMC4543231

[89] Gamis AS Alonzo TA Meshinchi S Sung L Gerbing RB Raimondi SC Gemtuzumab ozogamicin in children and adolescents with de novo acute myeloid leukemia improves event-free survival by reducing relapse risk: results from the randomized phase III Children’s Oncology Group trial AAML0531. J Clin Oncol (2014) 32:3021–32. 10.1200/JCO.2014.55.3628 25092781 PMC4162498

[90] Kenderian SS Ruella M Shestova O Klichinsky M Aikawa V Morrissette JJ CD33-specific chimeric antigen receptor T cells exhibit potent preclinical activity against human acute myeloid leukemia. Leukemia (2015) 29:1637–47. 10.1038/leu.2015.52 25721896 PMC4644600

[91] O’Hear C Heiber JF Schubert I Fey G Geiger TL. Anti-CD33 chimeric antigen receptor targeting of acute myeloid leukemia. Haematologica (2015) 100:336–44. 10.3324/haematol.2014.112748 25480499 PMC4349272

[92] Pizzitola I Anjos-Afonso F Rouault-Pierre K Lassailly F Tettamanti S Spinelli O Chimeric antigen receptors against CD33/CD123 antigens efficiently target primary acute myeloid leukemia cells in vivo. Leukemia (2014) 28:1596–605. 10.1038/leu.2014.62 24504024

[93] Tettamanti S Marin V Pizzitola I Magnani CF Giordano Attianese GM Cribioli E Targeting of acute myeloid leukaemia by cytokine-induced killer cells redirected with a novel CD123-specific chimeric antigen receptor. Br J Haematol (2013) 161:389–401. 10.1111/bjh.12282 23432359

[94] Mardiros A Dos Santos C McDonald T Brown CE Wang X Budde LE T cells expressing CD123-specific chimeric antigen receptors exhibit specific cytolytic effector functions and antitumor effects against human acute myeloid leukemia. Blood (2013) 122:3138–48. 10.1182/blood-2012-12-474056 24030378 PMC3814731

[95] Gill S Tasian SK Ruella M Shestova O Li Y Porter DL Efficacy against human acute myeloid leukemia and myeloablation of normal hematopoiesis in a mouse model using chimeric antigen receptor-modified T cells. Blood (2014) 123:2343–54. 10.1182/blood-2013-09-529537 24596416 PMC3983612

[96] Ritchie DS Neeson PJ Khot A Peinert S Tai T Tainton K Persistence and efficacy of second generation CAR T cell against the LeY antigen in acute myeloid leukemia. Mol Ther (2013) 21:2122–9. 10.1038/mt.2013.154 23831595 PMC3831035

[97] Lynn RC Poussin M Kalota A Feng Y Low PS Dimitrov DS Targeting of folate receptor beta on acute myeloid leukemia blasts with chimeric antigen receptor-expressing T cells. Blood (2015) 125:3466–76. 10.1182/blood-2014-11-612721 25887778 PMC4447861

[98] Grada Z Hegde M Byrd T Shaffer DR Ghazi A Brawley VS TanCAR: a novel bispecific chimeric antigen receptor for cancer immunotherapy. Mol Ther Nucleic Acids (2013) 2:e105. 10.1038/mtna.2013.32 23839099 PMC3731887

[99] Kloss CC Condomines M Cartellieri M Bachmann M Sadelain M. Combinatorial antigen recognition with balanced signaling promotes selective tumor eradication by engineered T cells. Nat Biotechnol (2013) 31:71–5. 10.1038/nbt.2459 23242161 PMC5505184

[100] Lanitis E Poussin M Klattenhoff AW Song D Sandaltzopoulos R June CH Chimeric antigen receptor T Cells with dissociated signaling domains exhibit focused antitumor activity with reduced potential for toxicity in vivo. Cancer Immunol Res (2013) 1:43–53. 10.1158/2326-6066.CIR-13-0008 24409448 PMC3881605

[101] Pui CH Behm FG Singh B Rivera GK Schell MJ Roberts WM Myeloid-associated antigen expression lacks prognostic value in childhood acute lymphoblastic leukemia treated with intensive multiagent chemotherapy. Blood (1990) 75:198–202. 2294988

[102] Wiersma SR Ortega J Sobel E Weinberg KI. Clinical importance of myeloid-antigen expression in acute lymphoblastic leukemia of childhood. N Engl J Med (1991) 324:800–8. 10.1056/NEJM199103213241204 1997852

[103] Putti MC Rondelli R Cocito MG Arico M Sainati L Conter V Expression of myeloid markers lacks prognostic impact in children treated for acute lymphoblastic leukemia: Italian experience in AIEOP-ALL 88-91 studies. Blood (1998) 92:795–801. 9680347

[104] Suggs JL Cruse JM Lewis RE. Aberrant myeloid marker expression in precursor B-cell and T-cell leukemias. Exp Mol Pathol (2007) 83:471–3. 10.1016/j.yexmp.2007.08.012 17963747

[105] Bhushan B Chauhan PS Saluja S Verma S Mishra AK Siddiqui S Aberrant phenotypes in childhood and adult acute leukemia and its association with adverse prognostic factors and clinical outcome. Clin Exp Med (2010) 10:33–40. 10.1007/s10238-009-0067-8 19779962

[106] Morgan RA Yang JC Kitano M Dudley ME Laurencot CM Rosenberg SA. Case report of a serious adverse event following the administration of T cells transduced with a chimeric antigen receptor recognizing ERBB2. Mol Ther (2010) 18:843–51. 10.1038/mt.2010.24 20179677 PMC2862534

[107] Di Stasi A Tey SK Dotti G Fujita Y Kennedy-Nasser A Martinez C Inducible apoptosis as a safety switch for adoptive cell therapy. New Engl J Med (2011) 365:1673–83. 10.1056/NEJMoa1106152 22047558 PMC3236370

[108] Zhao Y Moon E Carpenito C Paulos CM Liu X Brennan AL Multiple injections of electroporated autologous T cells expressing a chimeric antigen receptor mediate regression of human disseminated tumor. Cancer Res (2010) 70:9053–61. 10.1158/0008-5472.CAN-10-2880 20926399 PMC2982929

[109] Wang X Chang WC Wong CW Colcher D Sherman M Ostberg JR A transgene-encoded cell surface polypeptide for selection, in vivo tracking, and ablation of engineered cells. Blood (2011) 118:1255–63. 10.1182/blood-2011-02-337360 21653320 PMC3152493

